# Trans-Apical Transcatheter Aortic Valve Replacement in a Dialysis Patient with Systolic Heart Failure

**DOI:** 10.3390/medicina58030347

**Published:** 2022-02-24

**Authors:** Akira Oshima, Teruhiko Imamura, Hiroshi Onoda, Yohei Ueno, Ryuichi Ushijima, Mitsuo Sobajima, Nobuyuki Fukuda, Shigeki Yokoyama, Toshio Doi, Kazuaki Fukahara, Hiroshi Ueno, Koichiro Kinugawa

**Affiliations:** 1 The Second Department of Internal Medicine, University of Toyama, Toyama 930-0194, Japan; brother0917jp@gmail.com (A.O.); ohiro0203@gmail.com (H.O.); fef6ge@gmail.com (Y.U.); ryuryu0702@gmail.com (R.U.); soba1126@yahoo.co.jp (M.S.); nfukuda@med.u-toyama.ac.jp (N.F.); hueno@med.u-toyama.ac.jp (H.U.); kinugawa-tky@umin.ac.jp (K.K.); 2 Department of Surgery 1, Faculty of Medicine, University of Toyama, Toyama 930-0194, Japan; yoko1@med.u-toyama.ac.jp (S.Y.); tdoi@med.u-toyama.ac.jp (T.D.); fuka@med.u-toyama.ac.jp (K.F.)

**Keywords:** heart failure, valvular disease, aortic stenosis

## Abstract

Mortality and morbidity remain high following transcatheter aortic valve replacement (TAVR) in dialysis patients or those with low left ventricular ejection fraction. Therapeutic strategy for those with these comorbidities remains unestablished. We had a dialysis patient with peripheral artery disease and low left ventricular ejection fraction, who received successfully scheduled trans-apical TAVR following sufficient reverse remodeling by 3-month optimal medical therapy. Our strategy should be validated in a larger robust cohort.

## 1. Introduction

Dialysis patients often have systemic atherosclerosis, including aortic stenosis [1]. It is well-known that mortality and morbidity following aortic valve replacement (AVR) are high in such a cohort, predominantly due to advanced systemic atherosclerosis, impaired cardiac function, and multiple comorbidities [2,3,4,5]. Since February 2021, transcatheter aortic valve replacement (TAVR) for dialysis patients has been reimbursed in Japan, but practical experience has not yet been accumulated.

Another unsolved issue when treating severe aortic stenosis is reduced ejection fraction. Mortality and morbidity remain high following TAVR in patients with low left ventricular ejection fraction (LVEF) [6], and the optimal strategy to improve their clinical outcomes remains uncertain [7].

We encountered a dialysis patient with severe aortic stenosis and low LVEF, for whom we considered TAVR.

### 1.1. Case Report

#### 1.1.1. Medical History

A 76-year-old man depending on hemodialysis for 13 years, due to chronic glomerulonephritis with histories of non-disabling stroke and bilateral common iliac artery stentings, was admitted to the former institute complaining of dyspnea on exertion. Transthoracic echocardiography demonstrated a left ventricular ejection fraction of 25%, with maximum velocity in the aortic valve of 3.7 m/s, and an estimated aortic valve area of 0.50 cm^2^. Bilateral heart catheterization exhibited 90% stenosis in the mid left anterior descending artery, a peak-to-peak pressure gradient between the left ventricle and aorta of 46 mmHg, left ventricular end-diastolic pressure of 44 mmHg, and a cardiac index of 2.55 L/min/m^2^. The patient was referred to our institute for further treatment.

#### 1.1.2. Investigation Results

**Figure 1:**
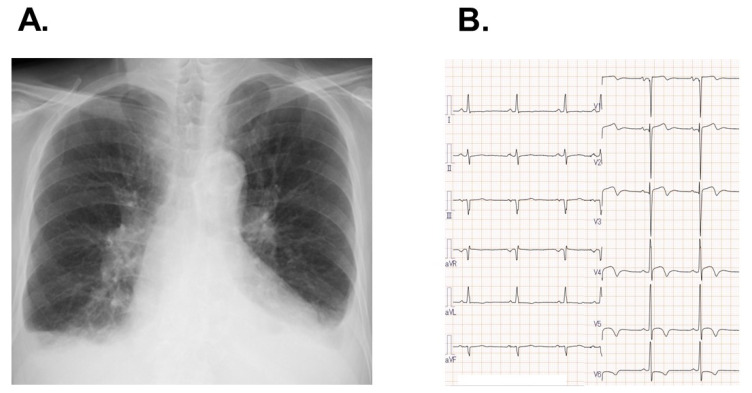
Chest X-ray displaying cardiomegaly, bilateral pulmonary congestion, and bilateral pleural effusion (**A**) and electrocardiogram showing heart rate of 64 bpm, sinus rhythm, and ST-segment depression in V5,6 (**B**) on admission.

The patient’s body height was 158 cm and his body weight was 46 kg. Systemic blood pressure was 142/84 mmHg and his pulse rate was 94 bpm. The estimated glomerular filtration ratio was 5.4 mL/min/1.73 m^2^ and plasma B-type natriuretic peptide was 8470 pg/mL. A chest X-ray displayed 61% of cardiothoracic ratio with bilateral mild pulmonary congestion (Figure 1A). An electrocardiogram depicted the sinus rhythm and slight ST depression in V5,6 (Figure 1B).

**Figure 2:**
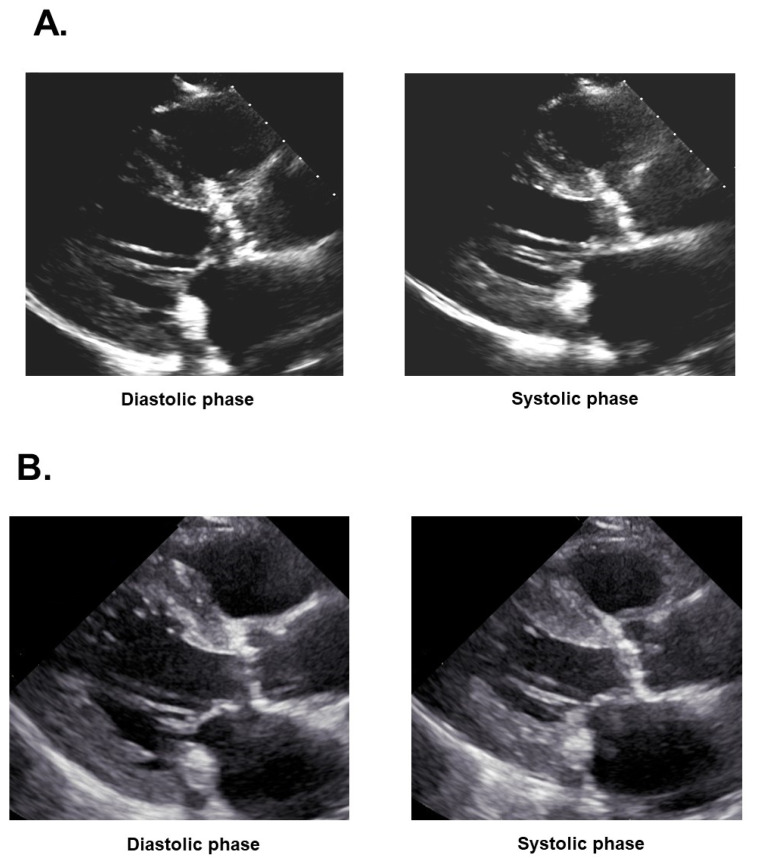
Transthoracic echocardiography (long-axis view) at diastolic/systolic phase on admission expressing left ventricular ejection fraction of 25% (**A**) and those following 3-month medical therapy showing left ventricular ejection fraction of 36% (**B**).

Transthoracic echocardiography revealed a left ventricular end-diastolic diameter of 44 mm and a left ventricular ejection fraction of 25% (Figure 2A). Maximum velocity in the aortic valve was 3.6 m/s, the estimated aortic valve area was 0.52 cm^2^, and the stroke volume index was 31 mL/m^2^. There was mild grade aortic regurgitation, mitral regurgitation, and tricuspid regurgitation. A dobutamine loading test demonstrated an incremental maximum velocity in the aortic valve from 3.37 m/s to 4.12 m/s, an incremental left ventricular ejection fraction from 31% to 40%, and the remaining aortic valve area from 0.77 cm^2^ to 0.66 cm^2^. Taken all together, the patient was diagnosed with low-flow low-gradient aortic stenosis.

**Figure 3:**
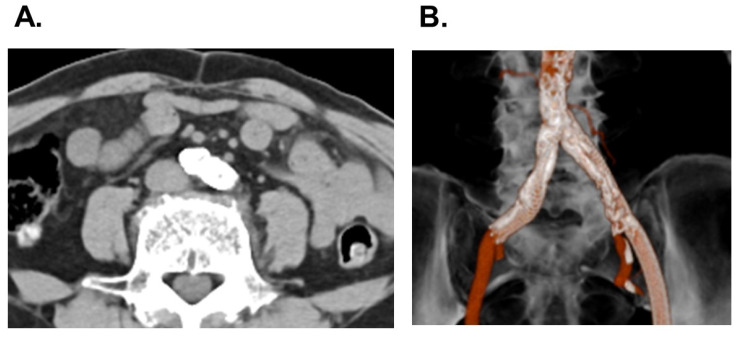
Abdominal computed tomography as a horizontal view (**A**) and three-dimensional view (**B**) on admission, possessing bilateral common iliac stents with small in-stent lumens.

Abdominal computed tomography showed stents in the bilateral common iliac arteries. However, in-stent lumens were very small: 1.2 mm in the right artery and 2.5 mm in the left artery (Figure 3A,B). The EURO II score was 6.1% and the STS score was 8.0%. The heart-valve team discussed therapeutic strategy and decided to perform a percutaneous coronary intervention and TAVR.

#### 1.1.3. Preparation for TAVR

**Figure 4:**
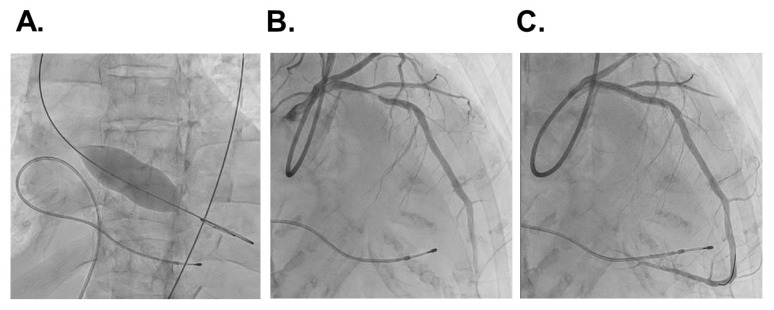
Balloon aortic valvuloplasty (**A**) and coronary angiography exhibiting significant stenosis at the mid left anterior descending artery (**B**) and those after successful percutaneous coronary intervention using drug-eluting stent (**C**).

On day 7, balloon aortic valvuloplasty (Figure 4A) and successive percutaneous coronary intervention were performed to the left anterior descending artery (Figure 4B,C). The trans-aortic valve peak-to-peak pressure gradient improved from 30 mmHg to 20 mmHg and the maximum velocity in the aortic valve improved from 3.57 m/s to 3.24 m/s.

**Figure 5:**
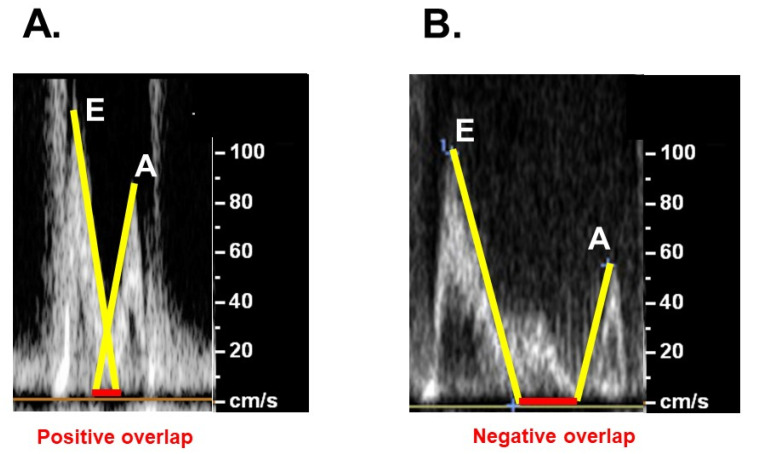
Trans-mitral inflow velocity focusing on the overlap between E-wave and A-wave using Doppler echocardiography on admission (**A**) and following heart rate modulation therapy using ivabradine (**B**). The two waves were overlapped in (**A**) and completely apart in (**B**).

Following the intervention, valsartan 40 mg/day was initiated to promote cardiac reverse remodeling. However, the patient’s systolic blood pressure decreased particularly during hemodialysis below 90 mmHg. Ivabradine 5.0 mg/day was initiated and his pulse rate decreased from 90 bpm to 62 bpm, and the overlap length between the E-wave and A-wave at trans-mitral flow echocardiography was minimized (Figure 5A,B) [8], accompanying the incremental systolic blood pressure to around 100 mmHg. Bisoprolol 0.3125 mg/day was initiated following the stabilization of blood pressure. Valsartan was decreased to 20 mg/day on non-hemodialysis days. Plasma B-type natriuretic peptide decreased to 4089 pg/mL. The patient was returned to the previously mentioned institute to further optimize medications to promote cardiac reverse remodeling and stabilize hemodynamics.

#### 1.1.4. Scheduled Operation

Three months later, the patient was again transferred to our institute to receive scheduled TAVR. On admission, his blood pressure was 146/68 mmHg and his pulse rate was 62 bpm. Left ventricular ejection fraction had improved from 25% to 36% (Figure 2B).

**Figure 6:**
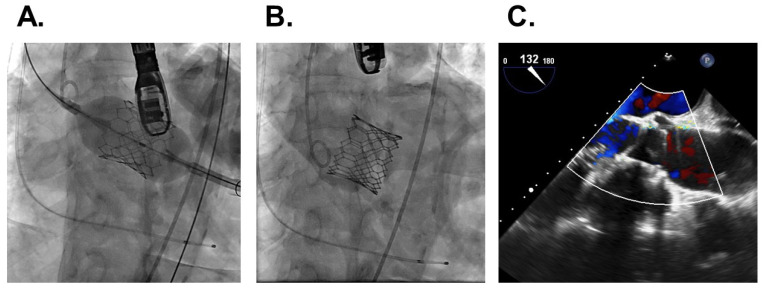
Trans-apical transcatheter aortic valve replacement. Aortic valve implantation (**A**), aortography without any peri-valvular leak (**B**), and transesophageal echocardiography displaying no peri-valvular leak (**C**).

Trans-apical TAVR was performed successfully without any peri-procedural complications (Figure 6A,B). Transesophageal echocardiography displayed no peri-valvular leak (Figure 6C). The patient was discharged on foot on post-operative day 11.

## 2. Discussion

A 76-year-old man with bilateral peripheral artery disease, and dependent on hemodialysis, successfully received scheduled trans-apical TAVR following cardiac reverse remodeling and balloon aortic valvuloplasty, with an improvement in left ventricular ejection fraction from 25% to 37% following 3-month optimal medical therapy, as well as complete revascularization of the coronary artery.

### 2.1. TAVR for Dialysis Patients

Dialysis patients often have aortic stenosis as one of the complications associated with systemic atherosclerosis [1]. Cumulative incidence of all-cause/sudden death is higher in dialysis patients following surgical AVR [2,5]. In a meta-analysis, a dependence on hemodialysis was an independent predictor of mortality following TAVR [9].

In Japan, an initial experience of TAVR in a dialysis patient was reported in 2015. A multicenter trial, including 28 Japanese dialysis patients, reported an 89.3%, 1-year survival rate following TAVR [10]. Given these findings, TAVR was reimbursed for the dialysis patients in February 2021.

TAVR in our patient was also challenging due to systemic atherosclerosis, including peripheral artery disease, stroke, coronary artery disease, and ischemic cardiomyopathy. Of note, our only option was to select the trans-apical approach, which was associated with higher risk than the femoral approach, given the small vascular diameters and severe calcification in other approach sites, including subclavian and ascending aorta, as often encountered in hemodialysis patients.

### 2.2. TAVR for Those with Low LVEF

A low LVEF is another unsolved issue that increases mortality and morbidity following TAVR [6]. Dialysis patients often have low LVEF due to various comorbidities including coronary artery disease and diabetes. Of note, post-TAVR insufficient cardiac unloading and residual tricuspid regurgitation are associated with heart failure recurrence [11]. Baseline LVEF < 30% is associated with worse clinical outcomes following trans-apical TAVR [12].

A therapeutic strategy for those with low LVEF receiving TAVR remains unestablished. Aggressive medications to unload the left ventricle using anti-hypertensive drugs and diuretics, as well as medications for heart failure including beta-blockers and renin-angiotensin–aldosterone system inhibitors, might facilitate reverse remodeling and prevent heart failure recurrence, as well as decreasing the risk of peri-procedural complications [11]. Heart rate modulation using ivabradine to minimize the overlap between the two trans-mitral inflow waves in the Doppler echocardiography might increase cardiac output and allow up-titration of other anti-heart failure medication, facilitating reverse remodeling [13].

In our patient, we postponed TAVR and waited three months to optimize medical therapy as detailed above, in addition to the balloon aortic valvuloplasty and complete coronary artery revascularization, all of which might have contributed to successive scheduled TAVR without any complications and achieving reverse remodeling. Of note, balloon aortic valvuloplasty was performed prior to the coronary intervention to prevent fatal ischemia during such intervention.

Appropriate candidates for this strategy remain unestablished. Those with an extremely unstable hemodynamic refractory to heart failure medications or those with less cardiac reserve might not be appropriate candidates. We continued optimal medical therapy for three months prior to TAVR, targeting sufficient reverse remodeling before aortic valve recoil, but appropriate pre-TAVR duration remains unknown. Further studies are warranted to validate such an aggressive medical therapy prior to TAVR for those with low LVEF.

## Data Availability

Data are available upon reasonable requests.
